# Supplementary material for: Using colour pattern edge contrast statistics to predict detection speed and success in triggerfish (Rhinecanthus aculeatus)
Source: J Exp Biol. 2022 Dec 9;225(23):jeb244677. doi: 10.1242/jeb.244677 (PMC9789405; doi:10.1242/jeb.244677)
Supplement: Supplementary information [file jexbio-225-244677-s1.pdf]

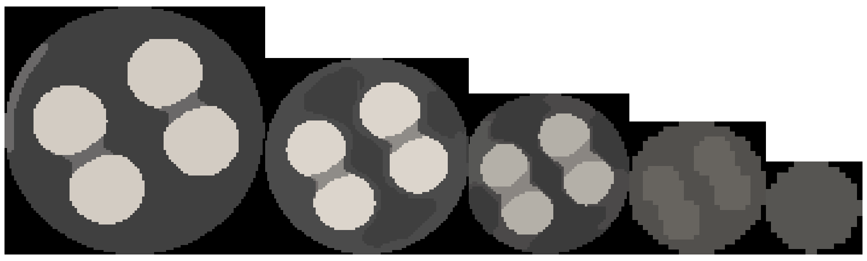

Fig. S1. Example of stimulus 1 in the achromatic treatment following acuity modelling & image resizing, RNL ranked filtering and RNL clustering over a modelled viewing distance of 2cm, 5cm, 10cm, 30cm and 50cm (left to right). Note the pronounced change in pattern & contrast over distance, including intermediate zones. RGB reconstructions based on the modelled cone stimulation of a triggerfish. Brightness and contrast adjusted for better visibility of pattern geometry.

Journal of Experimental Biology • Supplementary information

Table S1. Summary of all 17 parameters used in this study. For a detailed explanation of all parameters and their mathematical formulation, please see van den Berg et al. 2020b. The different analyses are colour coded throughout the manuscript.

Parameter Abbreviation	Parameter name	Parameter meaning
GabRat	Gabor Ratio	Average ratio of luminance edges at right angle to stimulus outline to luminance edges (measured as dbl stimulation) parallel with the stimulus outline
Lum.skew	LEIA luminance skewness	Skewness of histogram of all luminance edges measured in ΔS at the scale of an edge detecting receptive field
Lum.mean	LEIA mean luminance	Mean of luminance edges measured in ΔS at the scale of an edge detecting receptive field
Lum. kurt	LEIA luminance kurtosis	Kurtosis of histogram of all luminance edges measured in ΔS at the scale of an edge detecting receptive field
Lum.CoV	LEIA luminance coefficient of variation	Coefficient of variation (mean relative to standard deviation) of luminance edges measured in ΔS at the scale of an edge detecting receptive field
Lum.sd	LEIA luminance standard deviation	Standard deviation of luminance edges measured in ΔS at the scale of an edge detecting receptive field
Col.skew	LEIA chromatic skewness	Skewness of histogram of all chromatic edges measured in ΔS at the scale of an edge detecting receptive field
Col.mean	LEIA mean chromaticity	Mean of chromatic edges measured in ΔS at the scale of an edge detecting receptive field
Col.kurt	LEIA chromatic kurtosis	Kurtosis of histogram of all chromatic edges measured in ΔS at the scale of an edge detecting receptive field
Col.CoV	LEIA chromatic coefficient of variation	Coefficient of variation (mean relative to standard deviation) of chromatic edges measured

Journal of Experimental Biology • Supplementary information

		in ΔS at the scale of an edge detecting receptive field
Col.sd	LEIA chromatic standard deviation	Standard deviation of chromatic edges measured in ΔS at the scale of an edge detecting receptive field
BSA.BML	BSA weighted mean boundary luminance contrast	Abundance weighted mean of luminance edges measured as Michelson contrast of double cone stimulation between pattern elements
BSA.BsL	BSA standard deviation of boundary luminance contras	Standard deviation of luminance edges measured as Michelson contrast of double cone stimulation between pattern elements
BSA.BCVL	BSA coefficient of variation of boundary luminance contrast	Coefficient of variation of luminance edges measured as Michelson contrast of double cone stimulation between pattern elements
BSA.BMSsat	BSA weighted mean boundary saturation contrast	Abundance weighted mean of chromatic edges measured as Michelson contrast of chromatic ΔS between pattern elements
BSA.BsSsat	BSA standard deviation of boundary saturation contrast	Standard deviation of chromatic edges measured as Michelson contrast of chromatic ΔS between pattern elements
BSA.BCVSsat	BSA coefficient of variation of boundary saturation contrast	Coefficient of variation of chromatic edges measured as Michelson contrast of chromatic ΔS between pattern elements

Journal of Experimental Biology • Supplementary information

Table S2. All significant correlations tested for each viewing distance (D) (single parameter linear mixed effect regression models). Only parameters with significant (p) interactions are listed.

stimulus only				
Parm	D	*p*	F	%
BSA.BsL	5	<0.001	13.75	1.52
Lum.CoV	2	<0.001	12.53	1.40
BSA.BCVL	5	<0.001	12.67	1.39
BSA.BCVL	10	0.001	10.88	1.21
Lum.CoV	5	0.001	10.36	1.16
Col.mean	30	0.004	8.36	0.93
Col.mean	10	0.005	7.98	0.88
Lum.skew	2	0.007	7.32	0.82
Lum.mean	30	0.008	7.14	0.80
BSA.BML	10	0.008	7.01	0.78
Col.sd	2	0.009	6.87	0.76
Col.sd	10	0.011	6.55	0.73
Col.mean	2	0.011	6.51	0.72
Col.sd	5	0.011	6.44	0.71
Lum.CoV	10	0.012	6.28	0.70
Col.mean	5	0.013	6.18	0.68
Lum.skew	5	0.017	5.73	0.64
BSA.BML	30	0.017	5.69	0.64
Col.kurt	10	0.020	5.45	0.61
Lum.mean	2	0.020	5.42	0.61
BSA.BML	5	0.020	5.44	0.60
GabRat	2	0.023	5.17	0.58
Col.kurt	50	0.024	5.13	0.57
BSA.BMSsat	2	0.025	5.07	0.56
GabRat	5	0.028	4.86	0.54
Lum.kurt	2	0.028	4.84	0.54
BSA.BsSsat	30	0.033	4.58	0.51
Col.kurt	30	0.035	4.49	0.50
Col.kurt	5	0.037	4.39	0.49
Col.sd	30	0.048	3.92	0.43

stimulus vs. background				
Parm	D	*p*	F	%
Lum.CoV	5	<0.001	25.50	1.56
Lum.mean	2	<0.001	24.77	1.51
Lum.CoV	2	<0.001	23.38	1.43
Col.mean	30	<0.001	23.15	1.42
Lum.CoV	10	<0.001	22.40	1.37
Lum.mean	5	<0.001	20.75	1.26
BSA.BCVL	10	<0.001	20.42	1.24
Col.sd	10	<0.001	20.09	1.22
Col.sd	5	<0.001	20.06	1.22
Col.sd	2	<0.001	19.87	1.21
Col.sd	30	<0.001	19.51	1.20
BSA.BML	10	<0.001	19.26	1.18
BSA.BCVL	2	<0.001	16.14	0.98
Col.mean	10	<0.001	15.42	0.94
Lum.mean	30	<0.001	14.64	0.89
Col.mean	5	<0.001	14.60	0.89
BSA.BCVL	5	<0.001	14.26	0.87
Lum.skew	10	<0.001	14.11	0.86
Col.mean	2	<0.001	13.77	0.84
Lum.mean	10	<0.001	13.58	0.83
Col.CoV	2	<0.001	12.45	0.76
Col.CoV	5	0.001	11.64	0.71
Lum.sd	2	0.001	10.78	0.66
BSA.BMSsat	30	0.001	10.38	0.63
Lum.kurt	10	0.002	9.62	0.59
Lum.kurt	5	0.002	9.34	0.57
Col.CoV	30	0.003	8.89	0.54
BSA.BsL	10	0.003	8.77	0.53
BSA.BCVSsat	5	0.004	8.14	0.50
BSA.BsSsat	2	0.013	6.15	0.38
Lum.sd	30	0.013	6.14	0.38
Col.CoV	10	0.015	5.89	0.36
Col.skew	2	0.015	5.93	0.36
BSA.BsSsat	10	0.020	5.39	0.33
BSA.BML	5	0.022	5.23	0.32
Lum.sd	5	0.033	4.56	0.28
Lum.CoV	30	0.035	4.43	0.27
Col.kurt	2	0.039	4.25	0.26
Col.skew	5	0.044	4.05	0.25
Col.kurt	10	0.046	4.00	0.24
BSA.BCVL	30	0.047	3.96	0.24

stimulus only				
Parm	D	*p*	F	%
GabRat	10	<0.001	12.45	1.65
GabRat	5	0.006	7.58	1.00
BSA.BMSsat	30	0.007	7.35	0.97
Col.sd	2	0.008	7.15	0.94
Col.sd	5	0.010	6.64	0.88
BSA.BsSsat	10	0.013	6.25	0.84
Col.mean	30	0.016	5.89	0.78
Col.sd	10	0.027	4.93	0.65
Lum.mean	30	0.030	4.72	0.62
BSA.BsL	10	0.037	4.36	0.58
Lum.CoV	5	0.039	4.30	0.57
Col.sd	30	0.047	3.97	0.52
Lum.CoV	10	0.049	3.90	0.52

stimulus vs. background				
Parm	D	*p*	F	%
GabRat	10	<0.001	12.45	1.65
BSA.BsSsat	30	0.001	11.74	1.54
Lum.skew	10	0.006	7.67	1.01
GabRat	5	0.006	7.58	1.00
Lum.kurt	5	0.007	7.36	0.98
Lum.kurt	10	0.008	7.11	0.93
BSA.BsL	10	0.010	6.68	0.88
BSA.BsSsat	10	0.014	6.14	0.82
Lum.mean	2	0.013	6.15	0.81
Col.mean	30	0.026	5.00	0.66
BSA.BMSsat	30	0.032	4.64	0.61
Lum.CoV	5	0.046	3.98	0.53
Lum.mean	5	0.048	3.94	0.52
Lum.mean	30	0.049	3.89	0.52

stimulus only				
Parm	D	*p*	F	%
Lum.CoV	5	<0.001	25.91	1.58
Lum.CoV	2	<0.001	25.62	1.56
BSA.BCVL	5	<0.001	25.23	1.53
BSA.BCVL	10	<0.001	23.71	1.44
Col.mean	30	<0.001	23.50	1.44
Col.sd	30	<0.001	21.30	1.31
Lum.CoV	10	<0.001	21.03	1.28
BSA.BML	5	<0.001	20.61	1.25
Col.sd	10	<0.001	20.20	1.23
Col.sd	5	<0.001	20.11	1.22
Col.sd	2	<0.001	19.93	1.21
Col.CoV	2	<0.001	18.99	1.16
BSA.BML	10	<0.001	18.92	1.15
Lum.mean	2	<0.001	18.49	1.12
Col.CoV	5	<0.001	18.22	1.12
Col.mean	2	<0.001	18.05	1.10
Col.mean	5	<0.001	17.75	1.08
Lum.skew	2	<0.001	17.39	1.06
Col.mean	10	<0.001	17.46	1.06
Lum.skew	5	<0.001	16.48	1.01
Lum.mean	5	<0.001	15.14	0.92
BSA.BCVL	2	<0.001	14.24	0.86
Col.CoV	10	<0.001	13.55	0.83
Lum.skew	10	<0.001	12.80	0.78
Lum.kurt	2	<0.001	12.44	0.76
BSA.BsSsat	10	0.001	12.20	0.75
BSA.BsL	5	0.001	12.08	0.73
Lum.mean	30	0.001	11.95	0.73
BSA.BsSsat	2	0.001	11.59	0.71
Lum.kurt	5	0.001	11.23	0.69
BSA.BCVSsat	5	0.001	11.03	0.68
Lum.mean	10	0.001	10.87	0.66
BSA.BCVSsat	10	0.002	10.06	0.62
BSA.BML	2	0.002	9.46	0.57
BSA.BCVL	30	0.002	9.27	0.57
BSA.BsL	10	0.004	8.28	0.50
Lum.kurt	10	0.005	7.90	0.48
Lum.sd	30	0.006	7.65	0.47
BSA.BsSsat	5	0.007	7.30	0.45
Lum.sd	2	0.008	7.02	0.43
BSA.BMSsat	30	0.016	5.78	0.35
BSA.BCVSsat	2	0.022	5.26	0.32

stimulus vs. background				
Parm	D	*p*	F	%
Lum.CoV	5	<0.001	25.5	1.56
Lum.mean	2	<0.001	24.77	1.51
Lum.CoV	2	<0.001	23.38	1.43
Col.mean	30	<0.001	23.15	1.42
Lum.CoV	10	<0.001	22.4	1.37
Lum.mean	5	<0.001	20.75	1.26
BSA.BCVL	10	<0.001	20.42	1.24
Col.sd	10	<0.001	20.09	1.22
Col.sd	5	<0.001	20.06	1.22
Col.sd	2	<0.001	19.87	1.21
Col.sd	30	<0.001	19.51	1.2
BSA.BML	10	<0.001	19.26	1.18
BSA.BCVL	2	<0.001	16.14	0.98
Col.mean	10	<0.001	15.42	0.94
Lum.mean	30	<0.001	14.64	0.89
Col.mean	5	<0.001	14.6	0.89
BSA.BCVL	5	<0.001	14.26	0.87
Lum.skew	10	<0.001	14.11	0.86
Col.mean	2	<0.001	13.77	0.84
Col.mean	50	<0.001	13.61	0.84
Lum.mean	10	<0.001	13.58	0.83
Col.CoV	2	<0.001	12.45	0.76
Col.CoV	5	0.001	11.64	0.71
Lum.sd	2	0.001	10.78	0.66
BSA.BMSsat	30	0.001	10.38	0.63
Lum.kurt	10	0.002	9.62	0.59
Lum.kurt	5	0.002	9.34	0.57
Col.CoV	30	0.003	8.89	0.54
BSA.BsL	10	0.003	8.77	0.53
BSA.BCVSsat	5	0.004	8.14	0.5
BSA.BsSsat	2	0.013	6.15	0.38
Lum.sd	30	0.013	6.14	0.38
Col.CoV	10	0.015	5.89	0.36
Col.skew	2	0.015	5.93	0.36
BSA.BsSsat	10	0.02	5.39	0.33
BSA.BML	5	0.022	5.23	0.32
Lum.sd	5	0.033	4.56	0.28
Lum.CoV	30	0.035	4.43	0.27
Col.kurt	2	0.039	4.25	0.26
Col.skew	5	0.044	4.05	0.25
Col.kurt	10	0.046	4	0.24
BSA.BCVL	30	0.047	3.96	0.24

Journal of Experimental Biology • Supplementary information

Table S3. Summary of best models using pcr regression for each viewing distance (dist). The % of the variation in the data (% var), the % of explained variation in time to detection (% var resp) as well as the number of principal components (PC) are listed.

Combined					Chromatic				
**ROI**	**Dist**	**% var**	**% var resp**	**PCs**	**ROI**	**Dist**	**% var**	**% var resp**	**PCs**
Stim	2cm	62.66	3.18	3	Stimulus	2cm	23.55	0.11	1
Stim	5cm	47.84	3	2	Stimulus	5cm	99.79	5.05	13
Stim	10cm	98.18	5.16	11	Stimulus	10cm	91.14	2.67	6
Stim	30cm	99.95	5.73	15	Stimulus	30cm	96.21	6.33	8
Stim vs. BG	2cm	96.66	4.37	8	Stim vs. BG	2cm	99.3	2.82	9
Stim vs. BG	5cm	94.65	3.77	7	Stim vs. BG	5cm	94.85	2.37	7
Stim vs. BG	10cm	38.08	2.96	1	Stim vs. BG	10cm	38.72	0.35	1
Stim vs. BG	30cm	99.58	4.7	9	Stim vs. BG	30cm	99.96	4.2	9

Achromatic				
**ROI**	**Dist**	**% var**	**% var resp**	**PCs**
Stim	2cm	80.14	2.16	5
Stim	5cm	57.77	1.32	3
Stim	10cm	24.57	0.97	1
Stim	30cm	54.88	1.81	2
Stim vs. BG	2cm	92.93	3.32	6
Stim vs. BG	5cm	30.21	1.07	1
Stim vs. BG	10cm	49.53	0.93	2
Stimsvs. BG	30cm	96.62	2	6

Table S4. Summary of all stepwise regression analyses.

ROI	Treatment	Dist	Retained pattern statistics	% var expl
Stimulus	Achromatic	2cm	BSA.BML + Lum.CoV	2.45
Stimulus	Chromatic	2cm	Lum.mean	0.81
Stimulus	Achromatic	5cm	Lum.CoV	1.40
Stimulus	Chromatic	5cm	BSA.BCVSsat + BSA.BML + BSA.BMSsat + BSA.BsL + BSA.BsSsat + Col.CoV + Col.kurt + Col.mean + Col.sd	1.89
Stimulus	Achromatic	10cm	BSA.BCVL + BSA.BCVSsat + BSA.BsSsat + Col.skew + Lum.CoV	2.45
Stimulus	Chromatic	10cm	BSA.BMSsat + BSA.BsL + GabRat	2.67
Stimulus	Achromatic	30cm	BSA.BCVSsat + Col.CoV + Col.mean	2.92
Stimulus	Chromatic	30cm	BSA.BML + BSA.BMSsat + BSA.BsSsat	4.18
Stim vs. BG	Achromatic	2cm	BSA.BMSsat + Lum.CoV	2.58
Stim vs. BG	Chromatic	2cm	Col.sd	0.94
Stim vs. BG	Achromatic	5cm	BSA.BCVL + Col.skew + GabRat + Lum.CoV + Lum.kurt	3.53
Stim vs. BG	Chromatic	5cm	BSA.BCVSsat + Col.sd + GabRat + Lum.kurt	2.18
Stim vs. BG	Achromatic	10cm	BSA.BCVL + Col.kurt	1.98
Stim vs. BG	Chromatic	10cm	BSA.BCVSsat + BSA.BMSsat + Col.sd + GabRat + Lum.CoV + Lum.kurt	3.53
Stim vs. BG	Achromatic	30cm	BSA.BCVL + BSA.BCVSsat + BSA.BML + BSA.BsSsat + Col.kurt + Col.mean + Lum.mean	2.94
Stim vs. BG	Chromatic	30cm	BSA.BCVSsat + BSA.BMSsat + BSA.BsSsat + GabRat + Lum.mean	2.77
Stimulus	Combined	2cm	BSA.BCVSsat + BSA.BMSsat + Col.mean + Col.skew + GabRat + Lum.kurt + Lum.mean + Lum.skew	3.34
Stimulus	Combined	5cm	BSA.BCVSsat + BSA.BML + BSA.BsSsat + Col.mean + GabRat + Lum.CoV	2.27
Stimulus	Combined	10cm	BSA.BCVL + BSA.BCVSsat + BSA.BML + BSA.BMSsat + BSA.BsL + BSA.BsSsat + Col.CoV + Col.mean + Col.skew + GabRat + Lum.CoV + Lum.kurt + Lum.mean	3.41
Stimulus	Combined	30cm	BSA.BCVSsat + BSA.BML + BSA.BMSsat + BSA.BsSsat + Col.CoV + Col.mean	3.37
Stimulus	Combined	50cm	Col.mean	0.84
Stim vs. BG	Combined	2cm	BSA.BMSsat + Col.mean + Lum.CoV	2.38
Stim vs. BG	Combined	5cm	BSA.BsL + Col.mean + GabRat + Lum.mean	2.49
Stim vs. BG	Combined	10cm	BSA.BCVL + BSA.BML + BSA.BsL + Col.kurt + Col.mean + GabRat + Lum.mean	2.57
Stim vs. BG	Combined	30cm	BSA.BCVSsat + BSA.BML + BSA.BMSsat + BSA.BsL + BSA.BsSsat + Col.kurt + Col.sd + GabRat + Lum.mean + Lum.sd	3.06

Journal of Experimental Biology • Supplementary information
